# How Does Migration Background Affect COVID-19 Vaccination Intentions? A Complex Relationship Between General Attitudes, Religiosity, Acculturation and Fears of Infection

**DOI:** 10.3389/fpubh.2022.854146

**Published:** 2022-04-06

**Authors:** Manuel Holz, Jochen Mayerl, Henrik Andersen, Britta Maskow

**Affiliations:** Chemnitz University of Technology, Chemnitz, Germany

**Keywords:** vaccination intentions, migration, health inequalities, Structural Equation Modeling, acculturation, 5C model, religiosity, media consumption

## Abstract

**Objectives:**

The aim of the study is to investigate the relationship between migration background and COVID-19 vaccine intentions, exploring multiple mediation paths. We argue that the migrational and sociocultural background influences general attitudes toward health and political/public institutions. The effects of these general attitudes on vaccination intentions are mediated by fears of infection. Additionally, we analyze a migrant-only model including acculturation variables (years since migration, foreign and host country media consumption) and region of origin (European vs. Non-European). Design: The data (n = 1027) stem from an online access panel collected between March 15 and March 25, 2021. Quotas for gender and age were set according the online population of Germany. The use of an oversampling framework for first generation migrants resulted in a sample with 50% first generation migrants and 50% native Germans without migration background. Models were calculated using a Structural Equation Modeling approach.

**Results:**

Migration background both increases and decreases antecedents of vaccination intentions. Being a migrant increases positive antecedents like religiosity, which in turn positively influence general attitudes and thus fears of infection and vaccination intentions. But being a migrant has also a significant direct negative association with vaccination intentions, implying missing mediators. Increasing years since migration increase host country (German) media consumption and decrease consumption of media from the country of origin. Both media variables are positively associated with political trust and health consciousness. Additionally, European compared to Non-European migrants have less political trust, fear of personal infection and lower vaccination intentions on the whole.

**Conclusions:**

The study found that vaccination intentions can be understood by applying the proposed hypothetical structure. We found complex associations of the migration and sociocultural background and COVID-19 vaccination intentions, where antecedents of vaccination intentions are both increased and decreased by migration background and migration specific factors.

## Introduction

Since there is (so far) no mandatory vaccination program for SARS-Cov2 for the entire adult population in most of the countries worldwide, herd immunity by vaccination is largely dependent on voluntary decision making of the people. The extensive literature on the topic has delivered a repertoire of explanations for vaccination attitudes and intentions. Individuals negotiate their evaluation of the option to get vaccinated by the degree they trust the vaccine and its providers, perceptions of risks, costs and benefits, understanding and beliefs about health, the body and disease (1–6). The role of sociodemographics has been widely assessed and led to findings of differences in vaccination intention in categories of age, gender, education, income and ethnicity (7–10). However, broader attitudinal and belief-related factors in the context of vaccination have not been focused on yet. While it has been shown that more general attitudes and beliefs seldomly have direct effects on concrete health behavior (11), it can be assumed that during a totalizing event like the COVID-19 pandemic, more global belief structures influence health behavior like the intention to get vaccinated. We therefore seek to further explore the role of wider contextual aspects and their mediated effects on the intention to vaccinate against SARS-Cov2.

Migrants in several countries have been shown to be a vulnerable group during the COVID-19 pandemic, having higher infection prevalence and death tolls (4, 12) even after controlling for socioeconomic status (13). At the same time, most elemental resources to cope with this hazard, such as information, are very limited. In a quantitative analysis of governmental websites of member states of the European Union (14), it was shown that not one member state employed campaigns in the early days of the pandemic regarding information for health care in the languages spoken by the majority of migrants. While in the meantime there is some information available in target languages, one cannot speak yet of a nationwide provision.

The present study tries to understand and explain COVID-19 vaccination intentions with respect to differences in migration background. This article contributes to the literature in two ways: firstly, we explore the effect of migration background on individuals' risk assessment in the context of vaccination intention, and the role of health and political attitudes as potential mediators. We seek to understand social mechanisms of vaccination intention beyond socioeconomic and sociodemographic predictors. Secondly, we calculate a separate, migrant-only model including specific aspects of acculturation (years since migration, media consumption, region of origin) exploring explanations of potential differences in the vaccination attitudes compared to Germans without migration background.

## Vaccination Behavior and Migration: Theoretical Background

In this section we will outline briefly the way vaccination behavior can be understood through commonly applied behavioral models, but also through additional more general aspects. We further discuss the major elements of each in the context of migration and the consequences of having a migration background in Germany.

### 5C Model

To explain and understand vaccination behavior, behavioral models assume most fundamentally that individuals have the need to avoid illness and expect that specific action will prevent illness (15). From there, different perspectives have developed where one of the most frequent applied models is the 5C model (1), which makes individual psychological antecedents crucial to the prediction of vaccination intentions. These antecedents (the 5 “C‘s”) include: confidence (in the vaccine, the health care system, policy-makers), complacency (risk/threat perception of disease; need to employ health protective behavior), constraints (structural and psychological barriers), calculation (engagement in information seeking), collective responsibility (willingness to protect others). The model implies positive effects of confidence and responsibility; and negative effects of complacency, calculation and constraints on vaccination intentions.

Regarding *confidence*, it was found that higher levels of trust lead to higher acceptance or intention to get vaccinated. This could be found for trust in the vaccine (2, 4, 6, 16), science in general (16–18), health care providers (1, 2, 4), government (17–19) and media (18). In the case of migrants, Paul et al. (4) found lower levels of trust in the intentions of health care providers, whereas there is evidence for overall higher trust of migrants in political institutions like parliament and government (20, 21). Robertson et al. (6) showed that lower levels of confidence in ethnic minorities in the UK were still significant after controlling for sociodemographics.

Regarding *complacency*, individuals who perceive being at risk of infection with a disease in general or COVID-19 showed a higher acceptance of a vaccine (6, 9, 22–24). In the context of migration, it is important to acknowledge that higher numbers of infection, development of severe symptoms and death tolls in COVID-19 cases (4, 12, 25) can either imply a higher risk/threat perception (more infections, deaths, etc. → greater risk perception), or be the result of a lack of it (lack of risk perception → more infections, deaths, etc.).

*Constraints* in terms of, say, financial costs (3, 12) or expenditure of time (26) have been found to be significant in decreasing vaccine acceptance. Yet in a direct test of the 5C model, constraints measured as everyday work stress were not predictive of vaccine hesitancy (2). Relevant in the migration context are additional challenges for migrants such as living more frequently in high density living spaces (25) or working more frequently in unskilled labor (27, 28). These aspects constitute a higher exposure to health hazards (29) and a lack of resources to cope with them (30).

Regarding engagement of information seeking (*calculation*), results are still ambiguous. Hossain et al. (2) found positive effects of calculation (considering risks and benefits of vaccines) on the intention to vaccinate, whereas in Betsch et al. (22) the coefficient for this construct lacked statistical significance. In an attempt to explain the latter case, the authors suggest that a higher degree of information seeking might also lead to more exposure to false information. On the other hand, Strömbäck, Djerf-Pierre and Shehata (31) show that media consumption is positively associated with political trust. As mentioned earlier, there is evidence that migrants, especially in the beginning of the pandemic had less access to information and (traditional) media in their native language in the host country (14), which in turn increases susceptibility to misinformation.

Regarding *collective responsibility*, significant effects on vaccine acceptance were found in Betsch et al. (22) and in Murphy et al. (18). For migrants, it could be shown that individuals from typical migration countries to Germany score higher in collectivistic values than the general population of Germany (32).

For socioeconomic determinants, the literature delivers mixed results regarding vaccination attitudes and intentions. Socioeconomic status (as income and educational level) has been shown to be both a strong predictor of the antecedents of vaccination intentions (e.g., confidence and complacency) and vaccination intentions themselves (4). At the same time, ethnic minority status has been shown to have a robust negative effect on vaccination attitudes, even after controlling for demography and socioeconomic position (6, 7).

### Beyond Behavioral Models: Religiousness and Health Consciousness

Apart from individual influences as in other mentioned models, special attention is also given to religious beliefs (33). Religious beliefs can influence vaccination attitudes, e.g., by attempting to fit clinical reality within the theological framework and therefore increase vaccine refusal (33–35). On the other hand, religious beliefs are also linked to convictions to maintain one's health (36). For other health behaviors, significant effects were found for alcohol consumption (37, 38) and cancer screening (38), where higher religiosity was associated with an increase in health protective behavior. It can be assumed, due to higher attendance of religious services, that migrants show higher levels of religiosity than native Germans (39).

On a more general level, health consciousness has been shown to be predictive on a variety of health related measures. Positive associations have been found for diet and alcohol use (40–43), but also for health screening (44). Since there is no available research on the health consciousness of migrants in Germany, consumption behavior, as a result of health consciousness, will be informative for this case. There is evidence that migrants tend to have lower levels of tobacco and alcohol consumption per capita than native Germans (45, 46). Regarding diet, it has been shown that with increasing duration of residence in a European host country, Non-European migrants tend to reduce seafood and increase meat intake (47). The nutritional adaptation highlights the temporal component (i.e., the role of years since migration) of health consciousness and behavior.

### Migration Specific Factors: Acculturation and Region of Origin

Theoretically, increasing years since migration is accompanied with a higher degree of acculturation (48) translating into a higher degree of health care utilization when necessary, since the ability to express health concerns and handling administrative tasks in the target language improves (49–51). Immunization or vaccination use, however, yields quite ambiguous results. Higher levels of acculturation were actually found to be significant predictors of lower levels of vaccination in HPV (52) and MMR and Hepatitis B (53). On the other hand, a systematic literature review revealed that more recent (and therefore less acculturated) migrants tend to be undervaccinated (54). In the specific case of COVID-19 vaccination intention it was shown that acculturation was not a statistically significant predictor of vaccination intentions (55).

Of course, the outcomes of either acculturation or other aforementioned aspects are not uniform across all migrants. There are two dominant groups of migrants in Germany, namely (mostly Eastern and Southern) European and South West Asian (56). On a global level it could be shown that vaccine acceptance for a COVID-19 vaccine is lower, for example, among citizens in Turkey, with 66%, compared to Italy, with 77% (57). This raises the question of whether these differences in vaccination attitudes are maintained in the host country Germany, especially when socioeconomic and cultural backgrounds differ.

Socioeconomically, European migrants have shown to have more advantages than Non-European migrants in terms of labor market positions (27), net incomes (58), German language skills (59) and residential segregation (60). Further, it can be shown that European populations show lower levels in religious practice than Non-European populations (39).

## Proposed Model

We do not seek to replicate the 5C model 1:1, since the literature on the topic is already quite extensive. Moreover, we seek to propose a model where certain aspects of both behavioral and sociocultural aspects are put in relation to one another, so we try to shed light on multiple pathways to vaccination intention. In addition, we contribute to the ongoing discourse with a perspective that is less dependent on vaccine specific aspects and gives special attention to broader facets. We propose two models, where the first one (Model 1) is aimed at estimating the effect of migration and the relation to general and vaccine-specific factors, and a second model (Model 2) considering migrants only, examining migration-specific aspects within this framework. By doing so, we seek to find possible explanations in Model 2 for potentially differing vaccination intentions of migrants in Model 1.

**Figure 1:**
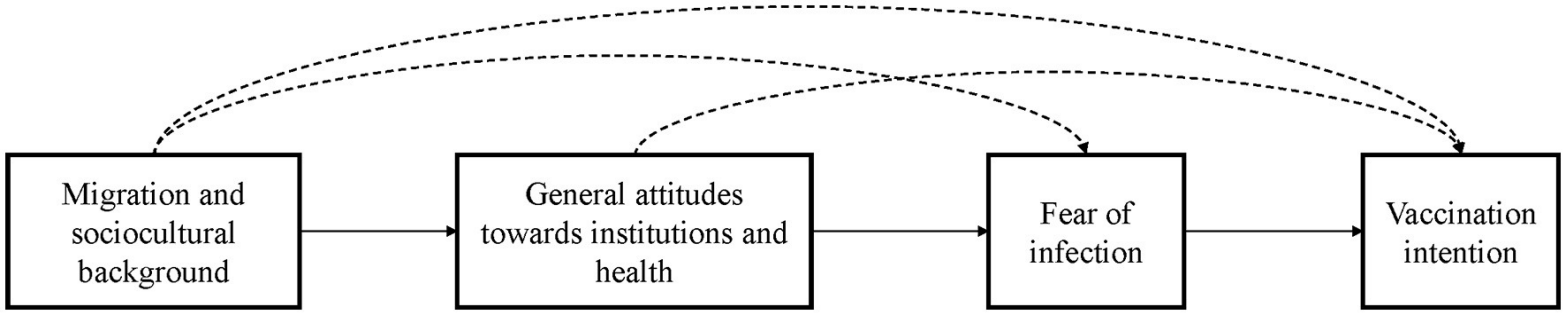
Hypothetical structure of vaccination intention.

For both cases (see Figure 1), we argue that the migration and sociocultural background mainly influences general attitudes toward political/public institutions and health. For Model 1, we specify the migration and sociocultural background as the migration status and the degree of religiosity. In Model 2, we specify this background as factors of acculturation and the region of origin. Acculturation includes years since migration and the type of media consumption (German vs. foreign media). For region of origin we use the indicator for European country of origin.

These factors determine attitudes toward institutions and health, which in turn are expected to have an effect on the intention to vaccinate, mediated by the fear of infection. On the level of vaccine specific factors, we use fear of infection and fear of transmission as the main mediators, where the former represents the individual and the latter the collectivistic aspect in the perceptions of fear and risk regarding COVID-19. Fear has been shown to be a mediator between risk assessment and health preventive behavior (61, 62). We argue that vaccination intention can be explained by applying this model, where migration and sociocultural background have no direct effects on the vaccination intention, but influence the outcome merely through the formulated mediation pattern.

To control for socioeconomic mechanisms, we include net income and educational level as additional predictors in all models. This approach allows us to identify explanations of vaccinations beyond socioeconomic position.

To explore the rather complex structure of health preventive behavior, we test each model in two variants: Firstly, we apply a strict hierarchical mediated structure (Model 1 and Model 2 without dashed lines in Figure 1). Secondly, we allow effects that empirically skip the strict hierarchical order (Model 1+ and Model 2+; including dashed lines in Figure 1).

## Data and Methods

### Data

We use cross-sectional data collected via a third-party online access panel provider (respondi AG). Respondents are randomly drawn from the database of the panel provider and incentivized by earning 50 Euro-Cents per 10-min interview time.

Interviews were available for smartphones and laptop/desktop computers. In order to increase representativity, we set quotas on age and gender according to the distribution in the online population in Germany (63). This resulted in 22.5% for age group 18–29, 41.2 % for 30–49 and 36.3% for 50–65 years. Gender quota was set to 49% for female and 51% for male respondents. We oversampled respondents with a first generation migration background, where quotas were put in place to ensure they made up 50% of the sample. Respondents with first generation migration background were defined as those who were not born in Germany, nor were either of their parents. The other 50% of the sample were respondents with no migration background (both they and both of their parents were born in Germany). The survey was further restricted to the population of age 18 to 65 residing in Germany. Data collection took place from March 15, 2021 until March 25, 2021.

### Measures

The main dependent variable is measured in line with Bendau et al. (2021) as intention to get vaccinated against COVID-19, given there is an opportunity in the following week (see Table 7 in the Appendix for exact wording). This item was measured using a 7-point rating scale (1: would definitely not get vaccinated, …, 7: would definitely get vaccinated).

As a main mediator we measure an individualistic and collectivistic aspect of fear of infection from COVID-19 (64), where respondents are asked to rate their degree of worry about becoming infected with COVID-19 (individualistic) and their degree of worry about infecting others (collectivistic). This item was measured on a 7-point rating scale (1: absolutely do not agree, …, 7: absolutely agree).

Trust in public institutions is measured according to the General German Social Survey (65). On a 7-point scale (1: no confidence at all, …, 7: very high confidence), respondents are asked to rate how high their confidence is in the federal government, the media, the police, the parliament and the health department.

For health consciousness we used three items from the health consciousness scale proposed by Pohle (66). On a 7-point rating scale, respondents were asked to rate their level of agreement to the statements: “health being the most important thing in life”, “worrying a lot when seeing reports of disease in the media” and “worrying a lot about one's own health”.

We use two items of the Frankfurt Acculturation Scale (67) to measure media usage of migrants. On a 7-point rating scale, respondents were asked to assess their level of agreement with the statement of whether they mainly consume media (television, news, radio etc.) from their own or their parents' country of origin, or media from Germany.

Regarding region of origin, European and Non-European respondents were coded according to the United Nations Statistics Division (68). Further, we computed years since migration from the year respondents first moved to Germany.

Additional covariates include: gender (male, female, diverse), age (in years), individual monthly net income (9-point scale, 1 ≤ 500 EUR, 9=more than 4000 EUR) and educational attainment (upper secondary degree and higher vs. lower secondary and lower).

### Analysis Strategy

We estimate the effects of migration background variables on vaccine intention, mediated by political trust and health consciousness, applying a Structural Equation Modeling approach to estimate path models with manifest and latent predictor variables. The use of a Full Information Maximum Likelihood (FIML) estimator allows us to retain more of the sample than with listwise deletion (69, 70). All analyses were conducted with R 4.1.1, lavaan package version 0.6–9.

## Results

### Descriptives

Table 1 shows the summary statistics of the data set. On average, native Germans without migration background have a bivariate, statistically significant (*p* = 0.000) higher vaccination intention (mean = 5.28, sd = 2.19) than first generation migrants (mean = 4.44, sd = 2.37). Looking further at the distribution, one can see that migrants have a higher share of the “not at all” response (21.1%) compared to Germans (14.2%). Also, migrants have a lower share of responses in the “definitely” category (34.9%) compared to native Germans without migration background (51.4%). When looking at overall tendencies, i.e., considering the first and last three categories together, we get the following picture: in overall accepting tendencies, migrants have a lower share (49.1%) than native Germans (68.7%). In overall rejecting tendencies, migrants have a higher share (31.8%) compared to native Germans (19.2%).

**Table 1:** Descriptive statistics.

**Variable**	**All** **(*n* = 1009)**	**Migrants** **(*n* = 477)**	**Native Germans** **(*n* = 532)**	***p*-value**
Vaccination intention, mean (sd)	4.87 (2.3)	4.44 (2.34)	5.28 (2.19)	0.000
Vaccination intention % (absolute)				
7 definitely	43.4 (439)	34.9 (165)	51.4 (268)	-
6	6.5 (66)	5.3 (25)	7.5 (39)	-
5	9.2 (93)	8.9 (42)	9.8 (51)	-
4	15.4 (156)	19.0 (90)	12.1 (63)	-
3	3.4 (34)	4.4 (21)	1.9(10)	-
2	4.6 (47)	6.3 (30)	3.1 (16)	-
1 not at all	17.5 (177)	21.1 (100)	14.2 (74)	-
Age, mean (sd)	42.97 (13.35)	41.56 (12.71)	44.13 (13.67)	0.000
Income, mean (sd)	4.85 (2.33)	4.84 (2.34)	4.85 (2.32)	0.940
Education% (absolute)				
Secondary degree	59.7 (592)	63.6 (288)	56.2 (292)	-
no secondary degree	40.3 (399)	36.4 (165)	43.8 (228)	-
Gender % (absolute)				
Female	49.9 (511)	53.7 (256)	47.1 (250)	-
Male	50.0 (510)	46.1 (220)	52.9 (281)	-
Diverse	0.1 (1)	0.2 (1)	-	-
Years since migration, mean (sd)	-	22.6 (16.38)	-	-
Region of origin % (absolute)				
Europe	-	63.3 (292)	-	-
Non-Europe	-	21.0 (97)	-	-
other	-	15.6 (72)	-	-

Further sociodemographics can be taken from the table (Table 1). It is important to note that in Model 2, we eliminated migrants who used the “other” category in country of origin since we are interested in the effect of region of origin and no conclusions can be drawn without knowledge of the region of origin.

### Multivariate Results

#### Measurement Models

Tables 3 and 4 (see Appendix) show fit measures and estimated coefficients for the measurement models of the latent constructs. For both models satisfactory fit can be observed in terms of the Comparative Fit Index (Model 1: 0.988, Model 2: 1.000), the Root Mean Square Error of Approximation (Model 1: 0.049, Model 2: 0.009) and the Standardized Root Mean Squared Error (Model 1: 0.027, Model 2: 0.020). All factor loadings for both models are significant at a 5% level. For the political trust construct, we included two error correlations to improve fit. Namely, the items used to measure trust in police and in the health department were shown to correlate above and beyond the latent factor, which is likely due to the concrete, “everyday” nature of these institutions^1^. The same goes for the items measuring trust in the media and in the government, which represent a more abstract, intangible dimension^2^. The item used to measure trust in parliament was eliminated from the latent construct due to a weak standardized factor loading (< 0.2). No cross-loadings were detectable.

#### Empirical Models

##### Model 1

Regarding model fit (see Table 2), the model performed decently with a CFI of 0.949 and a SRMR of 0.037. The RMSEA was, however, less than optimal at 0.059. Still, the upper 90% confidence interval (0.066) does not reach the 0.08 threshold suggested in the literature (71).

**Table 2:** Fit measures of empirical models.

**Model**	**Features**	**Chisq**	**df**	**p**	**RMSEA** **(90%-CI)**	**SRMR**	**CFI**
Model 1	All respondents, restricted	284.788	62	0.000	0.059 (0.052–0.066)	0.037	0.949
Model 1+	All respondents, additional paths	232.772	58	0.000	0.054 (0.047–0.062)	0.029	0.960
Model 2	Migrants only, restricted	175.429	88	0.000	0.051 (0.040–0.061)	0.037	0.951
Model 2+	Migrants only, additional paths	154.131	84	0.000	0.046 (0.035–0.058)	0.034	0.961

*RMSEA, Root Mean Square Error of Approximation, SRMR: Standardized Root Mean Squared Error*.

*CFI, Comparative FIt Index*.

##### Vaccination Intention

**Figure 2:**
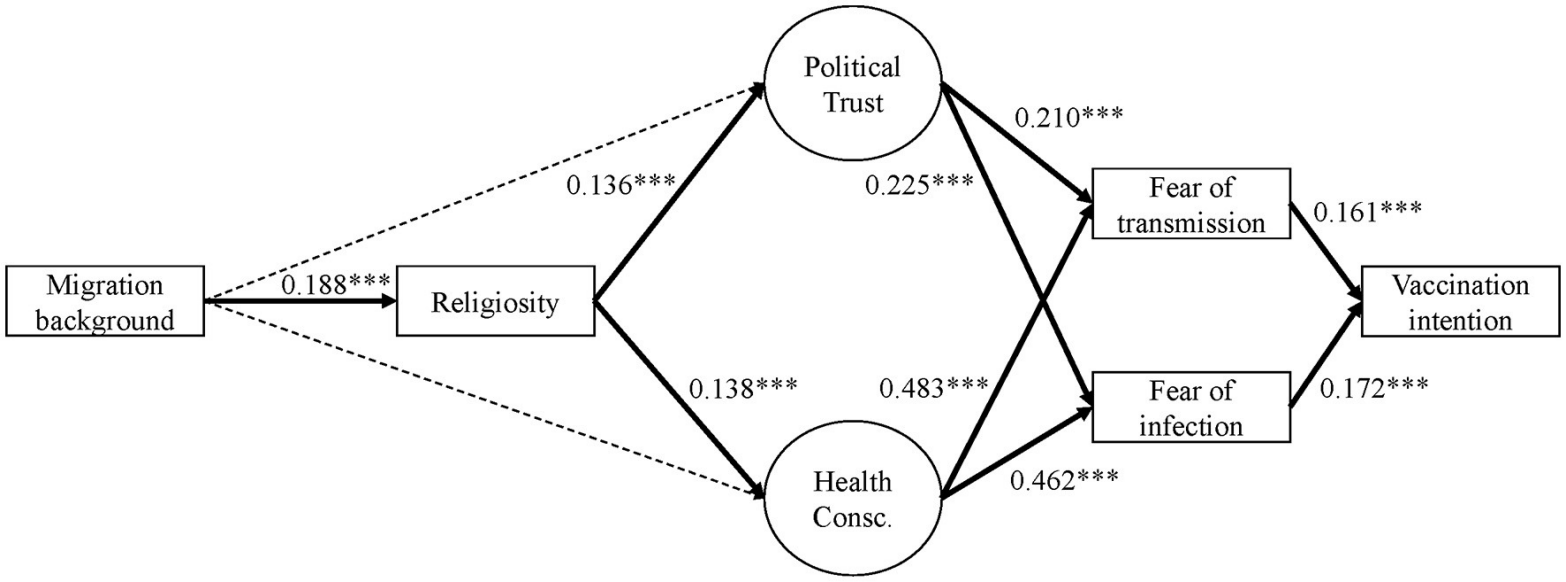
Empirical model 1, all respondents (standardized)^4^.

**Figure 3:**
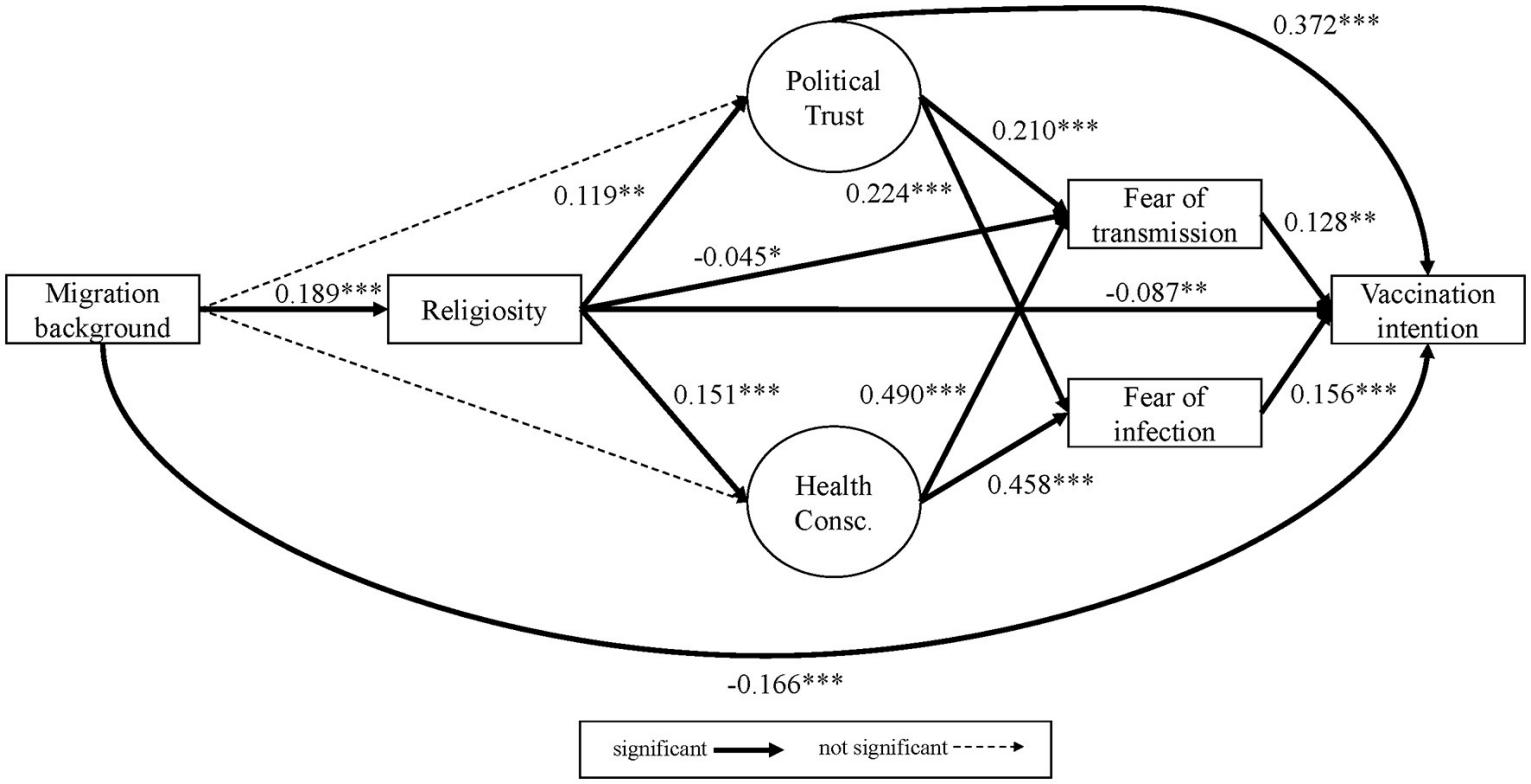
Empirical model 1+, all respondents (standardized) with additional paths as suggested by modification indices.

Looking at the results of Model 1 (Figure 2 and Table 5 in the Appendix), fear of infection and fear of transmission are positively associated with intention to vaccinate (*b* = 0.213, b^*^ = 0.161 and *b* = 0.192, b^*^ = 0.172). i.e., the more fearful one is of becoming infected or infecting others, the higher their willingness to get the vaccine. These effects remain quite stable in Model 1+ (see Figure 3 and the output for model 1+ in the online Appendix). Further, we find additional significant effects in Model 1+ for religiosity (direct negative effect: *b* = −0.112, b^*^ = −0.087) and political trust (direct positive effect: *b* = 0.773, b^*^ = 0.372). Most importantly, we observe a significant negative direct effect of being a first generation migrant on vaccination intention compared to native Germans without a migration background (*b* = −0,762; b^*^ = −0,166). The total effect of migration on vaccination intention (i.e., the addition of all possible direct and indirect pathways after controlling for socioeconomic and demographic predictors) is very small yet significant and positive in Model 1 (*b* = 0.027, SE = 0.008, b^*^ = −0.006, *p* = 0.001), but through the additional pathways in Model 1+, the total effect gains more substance and changes direction, mainly due to the direct negative effect (*b* = −0.780, SE = 0.123, b^*^ = −0.170, *p* = 0.000).

##### Fear of Infection and Fear of Transmission

Both political trust and health consciousness have significant positive effects on fear of infection and fear of transmission in models 1 and 1+ (Figures 2, 3). The standardized effects of political trust on both fear constructs are rather weak (about b^*^ = 0.21 to b^*^ = 0.22), whereas health consciousness shows a much stronger effect of about b^*^ = 0.46 to b^*^ = 0.49. That means, the higher the political trust and the higher the health consciousness, the higher the vaccination intention. In the extended model (Model 1+), another effect was found for religiosity, where an increase in the religiosity item decreases fear of transmission by *b* = −0.049^3^ points (b^*^ = −0.045).

##### Political Trust and Health Consciousness

First generation migrants do not differ significantly in political trust nor in health consciousness compared to native Germans without migration background in models 1 and 1+ (*p* > 0.1). But religiosity has a significant positive—but weak—effect on political trust (Model 1: b^*^ = 0.14; Model 1+: b^*^ = 0,12) as well as a weak positive effect on health consciousness (Model 1: b^*^ = 0,14; Model 1+: b^*^ = 0.15). Thus, people who perceive themselves as being more religious have a tendency to be more health conscious and have more trust in political and public institutions.

##### Religiosity

On average, migrants show a significantly higher level of religiosity (Model 1: *b* = 0.671, b^*^ = 0.188; Model 1+: b^*^ = 0.189) compared to native Germans.

##### Effects of Socioeconomic Position and Demography

For the socioeconomic predictors, we found siginificant positive effects of educational level on vaccination intentions (*b* = 0.701, se = 0.143, b^*^ = 0.150) and political trust (*b* = 0.276, se = 0.085, b^*^ = 0.122), and significant negative effects of education on health consciousness (*b* = −0.245, se = 0.089, b^*^ = −0.111) and religiosity (*b* = −0.421, se = 0.120, b^*^ = −0.066). Personal monthly net income had a significant direct effect on vaccination intentions, where higher levels of income were associated with higher levels of vaccination intentions (*b* = 0.062, se = 0.031, b^*^ = 0.063).

Men have on average higher levels of vaccination intentions than women (*b* = 0.526, se = 0.137, b^*^ = 0.114), less fear of transmission (*b* = −0.288, se = 0.111, b^*^ = −0.075) and lower levels of health consciousness (*b* = −0.199, se = 0.085, b^*^ = −0.092). Increasing age is associated with higher levels of vaccination intentions (*b* = 1.777, se = 0.557, b^*^ = 0.103) and less fear of transmission (*b* = −2.287, se = 0.438, b^*^ = −0.159).

##### Model 2

Regarding model fit, Model 2 (see Table 2), which includes only migrants, performs satisfactorily, as well. With a CFI of 0.951, a RMSEA of 0.051 (upper limit of 90% CI: 0.061) and a SRMR of 0.037, the indices are all within common thresholds.

##### Vaccination Intention

**Figure 4:**
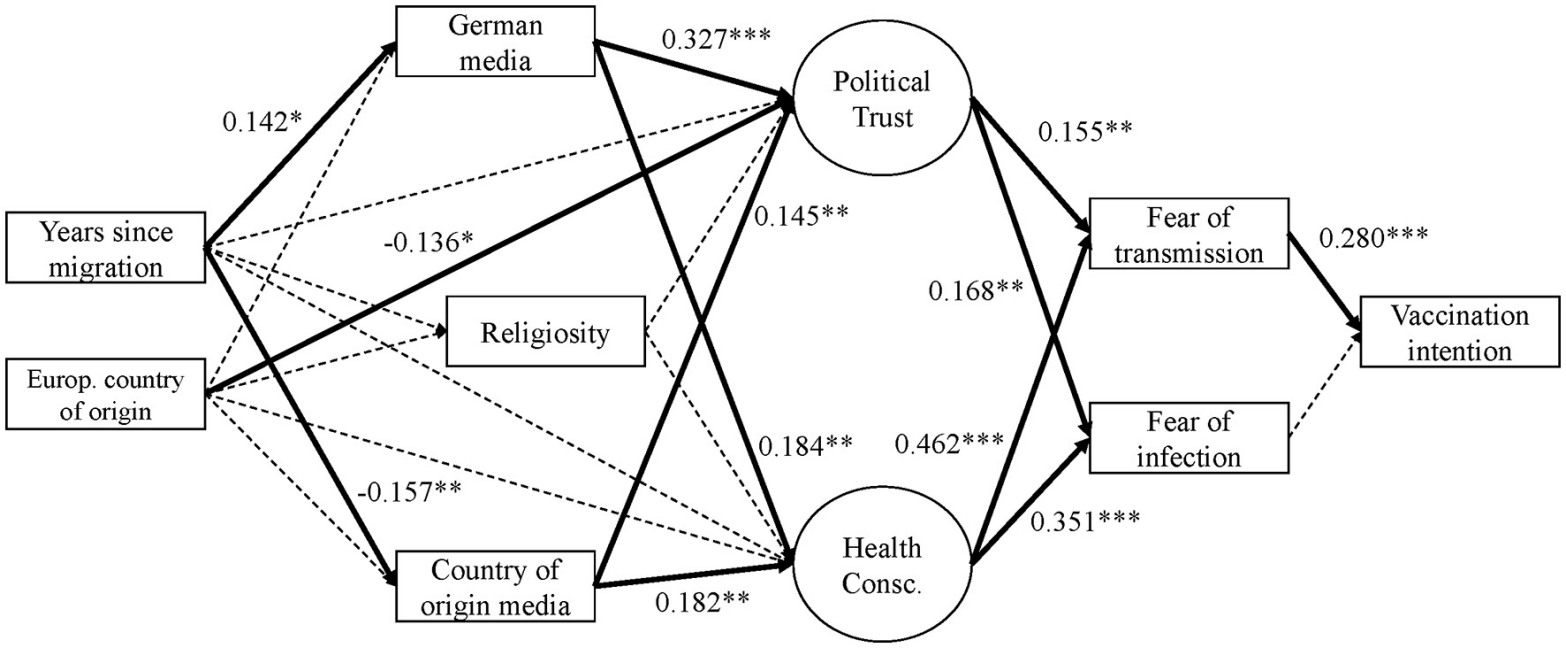
Empirical model 2—migrants only (standardized).

**Figure 5:**
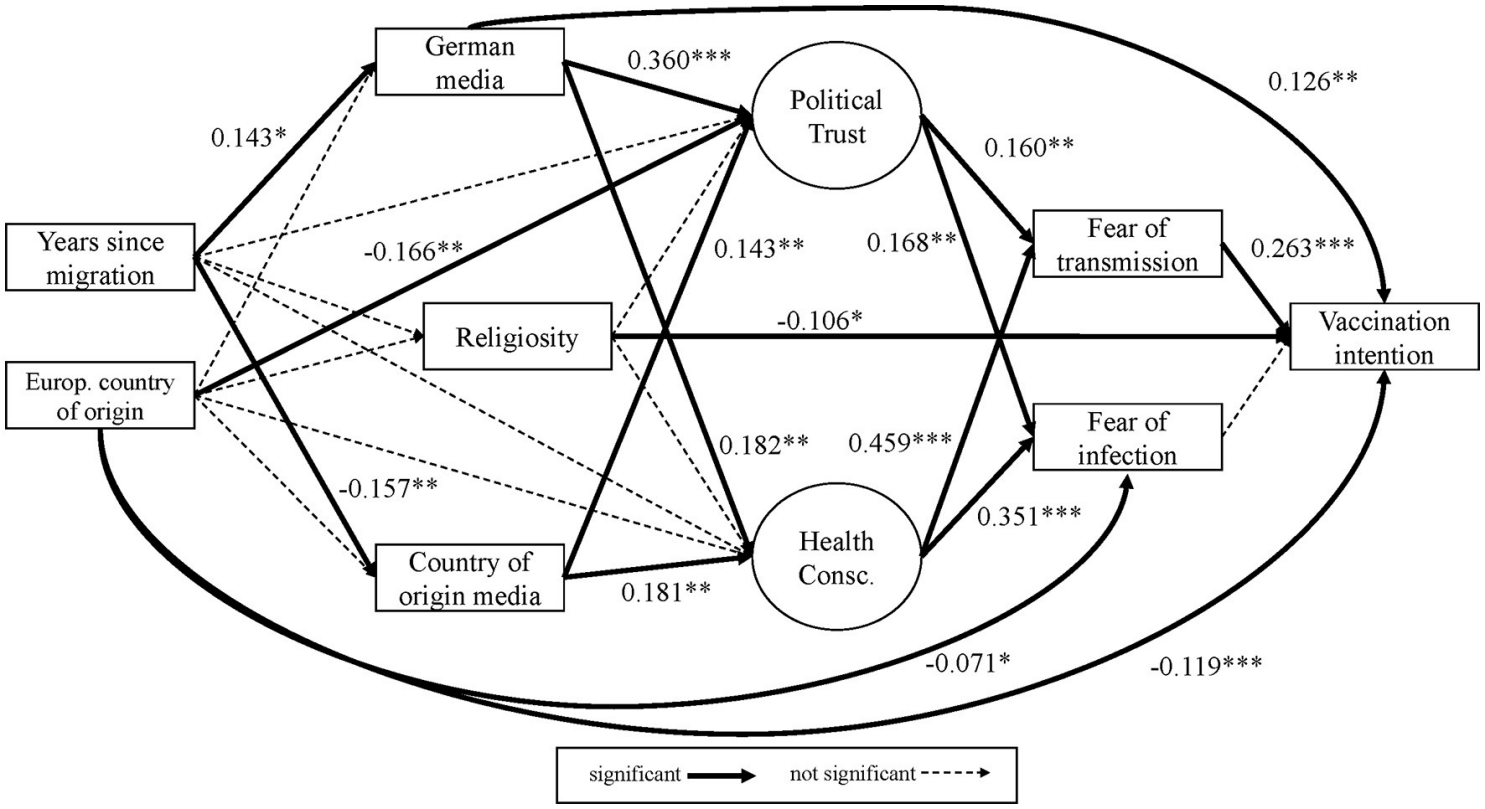
Empirical model 2+–migrants only (standardized) with additional paths as suggested by modification indices.

Looking at migrants only, we replicate the finding of Model 1 in that fear of transmission significantly increases vaccination intention (see Figures 4 and 5, as well Table 6 in the Appendix). A one point increase in fear of transmission increases vaccination intention by 0.346 points (Model 2: b^*^ = 0.280; Model 2+: b^*^ = 0,263). Interestingly, contrary to Model 1, fear of individual infection does not hold statistically significant its predictive power (*p* > 0.05).

Significant model improvement according to modification indices (see Model 2+) is achieved by including direct effects of German media consumption (b^*^ = 0.126), religiosity (b^*^ = −0.106), and European country of origin (b^*^ = −0.119). The latter means that European migrants, mostly from Eastern European countries such as Russia, Poland or Romania, have lower degrees of vaccine intention on average than migrants from South West Asia such as Turkey or Syria.

##### Fear of Infection and Fear of Transmission

Just as in Model 1, political trust and health consciousness are both positively and significantly associated with fear of infection and transmission. Again, the effect of health consciousness is much stronger (b^*^ of about 0.35 to 0.46) compared to political trust (b^*^ of about 0.16 to 0.17). Modification indices proposed the inclusion of European origin to directly predict fear of individual infection (Model 2+): scores for migrants with a European migration background are 0.313 lower, on average, than migrants with a non-European migration background in terms of fear of infection (b^*^ = −0.071).

##### Political Trust and Health Consciousness

Contrary to Model 1, religiosity does not significantly affect political trust (*p* > 0.05) and health consciousness (*p* > 0.05) in the migrant only models 2 and 2+.

Looking at factors affecting political trust, we find that consuming media from the country of origin enhances political trust (Model 2: b = 0.072, b^*^ = 0.145; Model 2+: b^*^=0.143). The same effect direction is found for consumption of German media (Model 2: *b* = 0.176, b^*^ = 327; Model 2+: b^*^ = 0.360). Years since migration has no direct effect on political trust (*p* > 0.1). A European migration background is negatively and significantly associated with political trust compared to a Non-European migration background (Model 2: *b* = −0.309, b^*^ = 0.136; Model 2+: *b* = −0.382, b^*^ = −0.166).

Health consciousness is determined by both consumption of media from the country of origin and from Germany with a weak effect of about b^*^ = 0.18. Neither years since migration (*p* > 0.1) nor region of origin (*p* > 0.1) predict health consciousness.

##### Media From Country of Origin and Media From Germany

In accordance with our expectations, media consumption can be predicted by years since migration. Each year since migration leads to an average decrease in the use of foreign media (b^*^ = −0.157 in models 2 and 2+). With every year spent in Germany, migrants tend to comsume more German media (b^*^ = 0.14 n models 2 and 2+). The other effects are not significant (*p* > 0.05).

##### Religiosity

Religiosity can neither be explained by years since migration (*p* > 0.1) nor by European country of origin (*p* > 0.1).

##### Effects of Socioeconomic Position and Demography

Educational level is associated positively with vaccination intentions (*b*=1.038, se = 0.242, b^*^ = 0.206) and negatively with religiosity (b = −0.812, se = 0.212, b^*^ = −0.204), whereas income showed no significant effect on any dependent variable.

Gender was only stastistically relevant in vaccination intentions, where men had higher levels of vaccination intentions than women (*b* = 0.694, se = 0.227, b^*^ = 0.146). Increased age was associated with higher levels of vaccination intentions (*b* = 3.147, se = 0.982, b^*^ = 0.164), less fear of transmission (*b* = −2.067, se = 0.801, b^*^ =- 0.133) and more consumption of German media (*b* = 2.084, se = 0.896, b^*^ = 0.140).

## Discussion

It is important to note that the intention to be vaccinated can be explained by the application of the hypothetical structure which we formulated in section 3. This is reflected in a sufficient model fit (see Table 2) for both Model 1 and Model 2, even without further loosening of correlational restrictions. Our findings contribute to the broad literature regarding the 5C Model and sociocultural perspectives in so far as we were able to formulate a “hierarchical” structure of the antecedents of vaccination intention. Vaccination intentions can therefore be understood as a result of effects of the migration and sociocultural background which are first mediated by general belief structures about health and institutions (comparable to confidence in the 5C perspective) and subsequently through fears of infection (both *complacency* and *collective responsibility)*.

It could be shown that the vaccine specific part of the model is in line with previous research.

Higher perceived risk and thus fear (*complacency* in the 5C Model) of COVID-19 infection predicted higher intention of a COVID-19 vaccination (6, 9, 23). It is thought that fear causes defensive reactions (72) and in the specific case of infectious diseases, the feeling of being threatened by disease is thought to change preventive behavior. Our findings contribute to the state of research, that this fear or risk assessment can be both of individual and collectivistic nature, where fear of either individual infection and infection of others by oneself leads to a higher impetus to vaccinate. However, this is only true if the whole population is the unit of analysis. Looking only at the migrant sample, individual fear of infection has no effect on vaccination intention, i.e., only the collectivistic component of fear is relevant in the internal negotiation of views toward vaccination. It is conceivable that a cultural orientation directed more toward collectivism makes collectivistic components of fear/risk assessment more relevant. This might be the case since populations from typical migration countries score higher in collectivism than the general population of Germany (32). Future research should investigate the mechanism of collectivistic orientation in the context of migration in more detail.

The overall lower vaccination intention of migrants (when compared to native Germans without migration background) as found in Model 1+ replicates the results of the already extensive research on the issue (4, 6, 18). Beyond the current state of research, we offer an additional explanatory path concerning the origin of the lack of vaccine acceptance, which goes beyond socioeconomic characteristics, bearing in mind that the effect of socioeconomic position could not be explained away entirely (in fact, education is one of the strongest predictors in all models).

We found, in line with current research (39), that migrants tend to have a higher level of religiosity. This effect has two consequences: on the one hand, there is a negative effect of religiosity on the collectivistic fear perceptions regarding COVID-19 infections, which in turn leads to a lower intention to vaccinate. We interpret this finding as a sign for the earlier mentioned notion (35), that religiosity can shift individual and environmental responsibility for disease and protective behavior to the realm of the divine and therefore away from personal agency. On the other hand, the heightened degree of migrant religiosity has a health protective dimension as well. The positive effect of religiosity increases two significant antecedents for vaccine intentions: trust in authorities (or *confidence*) and health consciousness. For the former effect Browne et al. (73) suggested that religiosity is associated with social conservatism and therefore with more acceptance of advice from established and conventional authorities. The latter effect is in concordance with the finding that religiosity can lead to more health preventive behaviors such as screening and adverse preferences for smoking and drinking (37, 38). Yosef (74) delivers a relevant explanation by pointing out that beliefs concerning purity and the perception of the body as a divine gift within abrahamic theology, might lead to better diet, more preventive screening and abstinence of hazardous substances.

To better understand underlying mechanisms within migration groups we employed Model 2 with a variety of migration specific indicators. As already shown in the literature, the role of acculturation is a complex one. We found positive effects of acculturation in the sense that increasing years since migration leads to an increase in German media consumption, which in turn is a mediator for trust (*confidence*), health consciousness and thus vaccination intention.

On the other hand, consumption of media from the country of origin decreases with years since migration. Yet, country of origin media consumption has a positive effect on political trust and health consciousness. In Mikolajczyk et al. (53) lower levels of acculturation are linked to higher vaccination rates. The authors argue that protection of Hepatitis B is higher in less acculturated respondents, because the prevalence is higher in the countries of origin and therefore risk perception is still elevated. Perhaps the positive association between health consciousness and political trust can be explained by foreign media reporting on the higher rates of COVID-19 fatalities in the countries of origin compared to the reporting in German media on the German situation. To draw actual conclusions regarding this issue, international comparisons of media content regarding COVID-19 fatalities, as well as media consumption patterns between migrant and native populations have to be studied.

Similarly to Model 1+, religiosity has a direct negative effect on vaccination intention, but no effect on political trust and health consciousness in the migrant-only sample. Evidence from Wong et al. (5) suggests that there is a growing concern among Muslim communities as to whether the COVID-19 vaccines are produced in line with *halal* requirements. Further, within Christian and anthroposophic communities Fournet et al. (34) identified a reason for undervaccination in the belief of destiny when it comes to disease as well as that a theologically oriented lifestyle alone has protective power against diseases. Further, educational level was identified to be the strongest predictor in migrant religiosity, while the role of acculturation seemed to be negligible. How exactly religion functions within different religious groups in the context of COVID-19 vaccination has yet to be studied in detail. The non-significance of religiosity on political trust, contrary to the all-respondents Model 1, can be interpreted as a sign that the social conservatism that accompanies religiosity (73) only applies to native Germans, since we only measured political trust in the host country institutions. This raises the question as to whether migrant religiosity in turn increases political trust in institutions of the country of origin. It is necessary to bear in mind that migration can already be a sign of distrust in the country of origin institutions, since the dissolution of social infrastructure and lacking economic opportunities are factors that drive people out of the country (75). With our data this question cannot be answered, however future research can make use of direct comparisons of different objects of trust in the context of religiosity.

Another interesting finding is the significantly lower vaccination intention of European compared to Non-European migrants. In Sallam (57) there is evidence that in an international comparison of COVID-19 vaccine acceptance the general populations of the Russian Federation (54.9%) and Poland (56.3%) score among the lowest, whereas Turkey (66 %), for example, is slightly more accepting, yet less so than Germany (70%). These findings correspond with the effects found in our study, yet the effects in Sallam (2021) relate to attitudes in the general population of the respective countries and not their emigrates to Germany. Apart from the general lower vaccination intention, our model provided a parallel path through political trust and fear of infection, where European migrants score lower in both measures. The reason why Europeans have lower trust in German authorities than Non-Europeans cannot be answered with our data and implicate missing mediators. Further research is needed to give plausible explanations.

## Strengths and Limitations

The strength of our study lies in the employment of a rigorous oversampling framework in the data collection process. In that way, the large share of first-generation migrants (50% of the sample) increases statistical power for the analysis and allows a more differentiated insight. Secondly, by using a Structural Equation Modeling framework, effect decomposition of multiple mediators on vaccine intentions helps to identify potentially differing paths to the outcome for different migration groups.

Beside our contribution, the generalizability of our results has some limitations. Firstly, due to the use of an online access panel sample, bias can be induced by varying degrees of participation probability in the general population. Further, access panel participation is already dependent on proficiency of the German language, which in turn leads to a skewed distribution toward more acculturated individuals. This is reflected in the distribution of the acculturation variables, where low scores in these measures were found only very rarely. To get a wider picture of the effects of acculturation, conventional face to face approaches and more survey translations are necessary to increase variance in acculturation indices.

Secondly, our sample is restricted solely to first generation migrants and Germans without any migration background. This was done in order to increase hard-to-reach groups like first generation migrants. In future studies, the inclusion of second generation migrants can shed light on more specific effects of acculturation.

Further bias is possible due to the sensitivity of the vaccination questions, since this issue has become a political issue as well. Opponents of vaccination might decline participation or respond in a socially desirable way. Approaches to reduce social desirability, like survey experiments, can be helpful in future studies.

Regarding the measures, it should be mentioned that we did not include attitudes toward vaccination or health beliefs in the context of culture or religion. Additionally, we did not investigate the role of media type, where it is conceivable that a potential reliance on a certain media type (e.g., print vs. television vs. online social media) can produce different effects on the outcome measures. Further research is needed to address the role of these factors.

Since we are using cross-sectional data in this study, no inferences can be made regarding causality, the results of our analysis are restricted to the nature of statistical associations.

## Conclusion

In this study we estimated two separate models in a SEM approach with vaccination intention as the outcome variable and socio-cultural factors as independent and mediator variables. We found that migration background is negatively associated with COVID-19 vaccination intention. Through multiple mediation pathways the model suggests positive and negative effects of religiosity on vaccination intention. Positive effects are mediated by political trust and health consciousness, whereas negative effects are mediated by fear of transmission. To further understand migrant specific factors, we estimated a second “migrant only” model with additional variables. We found, in coherence with the literature, complex associations regarding acculturation. Acculturation (measured in terms of years since migration) increases antecedents of vaccination intention such as German and foreign media consumption and thus political trust and health consciousness.

Moreover, we found a disparity between European and Non-European migrants, where the former show lower levels of political trust and lower overall vaccination intention than the latter.

Our results mirror frequent demands by public health scholars that campaigns regarding vaccination need to be tailored according to the target population's features and predispositions. First and foremost, it is necessary to offer all kinds of public health information in the target languages, such as Russian, Polish, Arabic, Turkish, Farsi or Kurmanji, especially for official government websites (14). In the dimension of content, interventions integrating religious teachings (74) and emphasizing collective utility of vaccination (22) can further motivate health seeking behavior. Face to face approaches can also help to increase vaccination acceptance in hard to reach communities (76). Regarding communication, it may be possible to increase trust in institutions by restricting the public discourse about COVID-19 to health care professionals and experts (77) and include media presenters from relevant migration groups (e.g., Poland, Russia, Turkey, Syria). In general, those with minority status (13), as well as asylum seekers (78) need to be included in the category of vulnerable groups by health agencies and public health institutions.

## Data Availability Statement

The datasets presented in this study can be found in online repositories. The names of the repository/repositories and accession number(s) can be found at: doi: 10.5281/zenodo.5795773.

## Author Contributions

MH, JM, HA, and BM had the idea of the research and conceptualized and designed the research. MH performed the statistical analysis, interpreted data, and drafted the initial manuscript. JM, HA, and BM supported the analysis and revised the manuscript critically. All authors contributed to the article and approved the submitted version.

## Funding

The publication of this article was funded by Chemnitz University of Technology and by the Deutsche Forschungsgemeinschaft (DFG, German Research Foundation) - 491193532.

## Conflict of Interest

The authors declare that the research was conducted in the absence of any commercial or financial relationships that could be construed as a potential conflict of interest.

## Publisher's Note

All claims expressed in this article are solely those of the authors and do not necessarily represent those of their affiliated organizations, or those of the publisher, the editors and the reviewers. Any product that may be evaluated in this article, or claim that may be made by its manufacturer, is not guaranteed or endorsed by the publisher.

## Footnotes

^1^The correlated items are “How much do you trust the health department?” and “How much do you trust the police”.

^2^The correlated items are “How much do you trust the federal government?” and “How much do you trust the media”.

^3^b stands for unstandardized coefficients, b^*^ for standardized coefficients.

^4^All effects controlled for education, gender, age, and income (see coefficients in the Appendix, Tables 5 and 6). This applies to all presented model.
