# Supplementary material for: How Does Migration Background Affect COVID-19 Vaccination Intentions? A Complex Relationship Between General Attitudes, Religiosity, Acculturation and Fears of Infection
Source: Front Public Health. 2022 Apr 6;10:854146. doi: 10.3389/fpubh.2022.854146 (PMC9019123; doi:10.3389/fpubh.2022.854146)
Supplement: Supplementary file 1 [file Table_1.docx]

Table 1: Model 1 - Measurement Model All respondents (n = 1027)

| **Latent construct** | **Indicator** | **Coefficient** | **SE** | **p-value** | **Standardized Coefficient** |
| --- | --- | --- | --- | --- | --- |
| Health Consciousness | Worried about health | 1.000 | - | - | 0.629 |
|  | Shocked by media reports about disease | 1.145 | 0.099 | 0.000 | 0.691 |
|  | Health most important in life | 0.923 | 0.075 | 0.000 | 0.582 |
| Political Trust | Federal government | 1.000 | - | - | 0.604 |
|  | Health deparment | 1.303 | 0.057 | 0.000 | 0.731 |
|  | Media | 1.226 | 0.174 | 0.000 | 0.736 |
|  | Police | 1.472 | 0.204 | 0.000 | 0.899 |
|  | Parliament | - | - | - | - |
| Error Correlations |  |  |  |  |  |
|  | Media ~ Police | -0.042 | 0.217 | 0.847 | -0.056 |
|  | Gov. ~ Health Dep. | 0.586 | 0.165 | 0.000 | 0.397 |
| Covariances |  |  |  |  |  |
|  | Health Consciousness ~  Political Trust | 0.278 | 0.053 | 0.000 | 0.286 |

**Fit measures:** Chi-Sq=38.082 df=11, p=0.000, CFI=0.988, RMSEA=0.049 (0.033- 0.066), SRMR=0.027

Table 2: Model 2 - Measurement Model Migrant only (n = 389)

| **Latent construct** | **Indicator** | **Coefficient** | **SE** | **p-value** | **Standardized Coefficient** |
| --- | --- | --- | --- | --- | --- |
| Health Consciousness | Worried about health | 1.000 | - | - | 0.681 |
|  | Shocked by media reports about disease | 0.981 | 0.128 | 0.000 | 0.659 |
|  | Health most important in life | 0.783 | 0.110 | 0.000 | 0.526 |
| Political Trust | Federal government | 1.000 | - | - | 0.539 |
|  | Health deparment | 1.388 | 0.104 | 0.000 | 0.716 |
|  | Media | 1.429 | 0.254 | 0.000 | 0.775 |
|  | Police | 1.752 | 0.298 | 0.000 | 0.960 |
|  | Parliament | - | - | - | - |
| Error Correlations |  |  |  |  |  |
|  | Media ~ Police | -0.178 | 0.287 | 0.535 | -0.383 |
|  | Gov. ~ Health Dep. | 0.712 | 0.184 | 0.000 | 0.435 |
| Covariances |  |  |  |  |  |
|  | Health Consciousness ~  Political Trust | 0.389 | 0.092 | 0.000 | 0.403 |

**Fit measures:** Chi-Sq=11.375 df=11, p=0.000, CFI=1.000, RMSEA=0.009 (0.000- 0.054), SRMR=0.020

Table 3 : Model 1 – All respondents (n = 1027)

| **Dependent Variable** | **Independent Variable** | **Unstandardized Coefficient** | **SE** | **p-value** | **Standardized Coefficient** | **R-squared** |
| --- | --- | --- | --- | --- | --- | --- |
| Vaccination Intention | Fear of infection | 0.213 | 0.048 | 0.000 | 0.172 | 0.186 |
|  | Fear of transmission | 0.192 | 0.048 | 0.000 | 0.161 |  |
|  | Education | 0.701 | 0.143 | 0.000 | 0.150 |  |
|  | Gender male | 0.526 | 0.137 | 0.000 | 0.114 |  |
|  | Income | 0.062 | 0.031 | 0.045 | 0.063 |  |
|  | Age | 1.777 | 0.557 | 0.001 | 0.103 |  |
| Fear of infection | Political Trust | 0.376 | 0.057 | 0.000 | 0.225 | 0.327 |
|  | Health Consciousness | 0.792 | 0.069 | 0.000 | 0.462 |  |
|  | Education | -0.007 | 0.111 | 0.949 | -0.002 |  |
|  | Gender male | -0.163 | 0.104 | 0.118 | -0.044 |  |
|  | Income | 0.004 | 0.023 | 0.868 | 0.005 |  |
|  | Age | 0.716 | 0.407 | 0.079 | 0.052 |  |
| Fear of transmission | Political Trust | 0.363 | 0.060 | 0.000 | 0.210 | 0.366 |
|  | Health Consciousness | 0.859 | 0.073 | 0.000 | 0.483 |  |
|  | Education | 0.012 | 0.118 | 0.922 | 0.003 |  |
|  | Gender male | -0.288 | 0.111 | 0.009 | -0.075 |  |
|  | Income | 0.045 | 0.025 | 0.070 | 0.054 |  |
|  | Age | -2.287 | 0.438 | 0.000 | -0.159 |  |
| Political Trust | Migrant (Ref.: Native) | 0.114 | 0.075 | 0.128 | 0.051 | 0.042 |
|  | Religiosity | 0.085 | 0.021 | 0.000 | 0.136 |  |
|  | Education | 0.276 | 0.085 | 0.001 | 0.122 |  |
|  | Gender male | 0.092 | 0.079 | 0.246 | 0.041 |  |
|  | Income | 0.014 | 0.018 | 0.447 | 0.028 |  |
|  | Age | -0.180 | 0.314 | 0.566 | -0.022 |  |
| Health consciousness | Migrant (Ref.: Native) | 0.109 | 0.082 | 0.185 | 0.050 | 0.053 |
|  | Religiosity | 0.084 | 0.023 | 0.000 | 0.138 |  |
|  | Education | -0.245 | 0.089 | 0.006 | -0.111 |  |
|  | Gender male | -0.199 | 0.085 | 0.019 | -0.092 |  |
|  | Income | -0.021 | 0.019 | 0.266 | -0.046 |  |
|  | Age | 0.583 | 0.340 | 0.087 | 0.072 |  |
| Religiosity | Migrant (Ref.: Native) | 0.671 | 0.111 | 0.000 | 0.188 | 0.046 |
|  | Education | -0.241 | 0.120 | 0.044 | -0.066 |  |
|  | Gender male | -0.219 | 0.115 | 0.056 | -0.062 |  |
|  | Income | 0.027 | 0.026 | 0.293 | 0.035 |  |
|  | Age | -0.556 | 0.450 | 0.217 | -0.042 |  |

Table 4 : Model 2 – Migrants only (n = 389)

| **Dependent Variable** | **Independent**  **Variable** | **Unstandardized Coefficient** | **SE** | **p-value** | **Standardized Coefficient** | **R-squared** |
| --- | --- | --- | --- | --- | --- | --- |
| Vaccination Intention | Fear of infection | 0.131 | 0.077 | 0.090 | 0.105 | 0.227 |
|  | Fear of transmission | 0.346 | 0.077 | 0.000 | 0.280 |  |
|  | Education | 1.038 | 0.242 | 0.000 | 0.206 |  |
|  | Gender male | 0.694 | 0.227 | 0.002 | 0.146 |  |
|  | Income | -0.057 | 0.051 | 0.267 | -0.056 |  |
|  | Age | 3.147 | 0.982 | 0.001 | 0.164 |  |
| Fear of infection | Political trust | 0.324 | 0.118 | 0.006 | 0.168 | 0.210 |
|  | Health consciousness | 0.587 | 0.130 | 0.000 | 0.351 |  |
|  | Education | -0.045 | 0.199 | 0.820 | -0.011 |  |
|  | Gender male | -0.137 | 0.181 | 0.449 | -0.036 |  |
|  | Income | -0.016 | 0.040 | 0.682 | -0.020 |  |
|  | Age | 0.898 | 0.763 | 0.239 | 0.058 |  |
| Fear of transmission | Political trust | 0.302 | 0.117 | 0.010 | 0.155 | 0.300 |
|  | Health consciousness | 0.781 | 0.124 | 0.000 | 0.462 |  |
|  | Education | 0.011 | 0.207 | 0.959 | 0.003 |  |
|  | Gender male | -0.180 | 0.189 | 0.340 | -0.047 |  |
|  | Income | 0.074 | 0.041 | 0.073 | 0.091 |  |
|  | Age | -2.067 | 0.801 | 0.010 | -0.133 |  |
| Political trust | Media country of origin | 0.072 | 0.026 | 0.006 | 0.145 | 0.163 |
|  | Media Germany | 0.176 | 0.031 | 0.000 | 0.327 |  |
|  | Years since migration | -0.132 | 0.412 | 0.748 | -0.020 |  |
|  | European (Ref.: Non-European) | -0.309 | 0.119 | 0.010 | -0.136 |  |
|  | Religiosity | 0.051 | 0.028 | 0.065 | 0.097 |  |
|  | Education | 0.228 | 0.124 | 0.066 | 0.109 |  |
|  | Gender male | 0.111 | 0.111 | 0.318 | 0.056 |  |
|  | Income | -0.035 | 0.024 | 0.149 | -0.082 |  |
|  | Age | 0.128 | 0.512 | 0.802 | 0.016 |  |
| Health consciousness | Media country of origin | 0.105 | 0.035 | 0.003 | 0.182 | 0.100 |
|  | Media Germany | 0.114 | 0.038 | 0.003 | 0.184 |  |
|  | Years since migration | 0.691 | 0.544 | 0.204 | 0.090 |  |
|  | European (Ref.: Non-European) | -0.030 | 0.155 | 0.849 | -0.011 |  |
|  | Religiosity | 0.047 | 0.037 | 0.203 | 0.078 |  |
|  | Education | -0.121 | 0.159 | 0.445 | -0.050 |  |
|  | Gender male | -0.104 | 0.149 | 0.486 | -0.046 |  |
|  | Income | -0.033 | 0.032 | 0.296 | -0.069 |  |
|  | Age | 0.595 | 0.684 | 0.384 | 0.065 |  |
| Media country of origin | Years since migration | -2.090 | 0.794 | 0.009 | -0.157 | 0.041 |
|  | European (Ref.: Non-European) | -0.430 | 0.227 | 0.058 | -0.095 |  |
|  | Education | -0.209 | 0.223 | 0.347 | -0.050 |  |
|  | Gender male | -0.051 | 0.212 | 0.810 | -0.013 |  |
|  | Income | -0.032 | 0.046 | 0.483 | -0.038 |  |
|  | Age | -0.079 | 0.975 | 0.936 | -0.005 |  |
| Media Germany | Years since migration | 1.755 | 0.729 | 0.016 | 0.142 | 0.060 |
|  | European (Ref.: Non-European) | 0.096 | 0.209 | 0.646 | 0.023 |  |
|  | Education | 0.073 | 0.208 | 0.725 | 0.019 |  |
|  | Gender male | -0.030 | 0.195 | 0.878 | -0.008 |  |
|  | Income | 0.017 | 0.043 | 0.695 | 0.022 |  |
|  | Age | 2.084 | 0.896 | 0.020 | 0.140 |  |
| Religiosity | Years since migration | -0.574 | 0.754 | 0.447 | -0.045 | 0.048 |
|  | European (Ref.: Non-European) | -0.333 | 0.217 | 0.125 | -0.077 |  |
|  | Education | -0.812 | 0.212 | 0.000 | -0.204 |  |
|  | Gender male | -0.100 | 0.202 | 0.619 | -0.027 |  |
|  | Income | 0.020 | 0.043 | 0.649 | 0.024 |  |
|  | Age | -0.905 | 0.934 | 0.333 | -0.060 |  |

Table 5: Question wording

| **Construct** | **Item (own translation from German)** | **Response Scale** |
| --- | --- | --- |
| Vaccination intention | How would you decide, if you had the opportunity to get vaccinated against COVID-19 next week? | 1: totally refuse to vaccinate  7: definitely vaccinate |
| Fear of infection | I fear to become infected with Corona | 1: totally disagree …  7: totally agree |
| Fear of transmission | I fear to transmit the virus to friends and relatives | 1: totally disagree …  7: totally agree |
| Political trust | How much do you trust the federal government? | 1: not at all  7: a lot |
|  | How much do you trust the health departement? | 1: not at all  7: a lot |
|  | How much do you trust the media? | 1: not at all  7: a lot |
|  | How much do you trust the police? | 1: not at all  7: a lot |
|  | How much do you trust the parliament? | 1: not at all  7: a lot |
| Health consciousness | I am worried about my health | 1: totally disagree …  7: totally agree |
|  | I am very shocked when there are reports about serious diseases in the media | 1: totally disagree …  7: totally agree |
|  | There is nothing more important in life than thinking about your health | 1: totally disagree …  7: totally agree |
| Religiosity | Would you say about yourself that you are rather religious or rather not religious? | 1: not religious at all  7: very religious |
| Migration background | Were you born in the territory of today’s Germany (also former GDR)? | yes/no |
|  | Were your parents born in the territory of today’s Germany (also former GDR)? | Yes both/ Just one parent/ No, neither |
| German media consumption | I use German media (tv, radio, newspaper, magazines etc.) | 1: totally disagree …  7: totally agree |
| Country of origin media consumption | I use media from my/ my parents’ country of origin (tv, radio, newspaper, magazines etc.) | 1: totally disagree …  7: totally agree |
| Years since migration | Since when do you live in Germany (also former GDR) | numeric |
| European country of origin | In which country were you born in? | nominal |
